# Antibiotic-resistant bacteria and gut microbiome communities associated with wild-caught shrimp from the United States versus imported farm-raised retail shrimp

**DOI:** 10.1038/s41598-021-82823-y

**Published:** 2021-02-08

**Authors:** Laxmi Sharma, Ravinder Nagpal, Charlene R. Jackson, Dhruv Patel, Prashant Singh

**Affiliations:** 1 Department of Nutrition, Food and Exercise Sciences, Florida State University, Tallahassee, FL 32306 USA; 2 Bacterial Epidemiology and Antimicrobial Resistance Research Unit, U.S. Department of Agriculture Agricultural Research Service, Athens, GA USA; 3 Department of Biological Sciences, Florida State University, Tallahassee, FL USA

**Keywords:** Food microbiology, Antimicrobial resistance

## Abstract

In the United States, farm-raised shrimp accounts for ~ 80% of the market share. Farmed shrimp are cultivated as monoculture and are susceptible to infections. The aquaculture industry is dependent on the application of antibiotics for disease prevention, resulting in the selection of antibiotic-resistant bacteria. We aimed to characterize the prevalence of antibiotic-resistant bacteria and gut microbiome communities in commercially available shrimp. Thirty-one raw and cooked shrimp samples were purchased from supermarkets in Florida and Georgia (U.S.) between March–September 2019. The samples were processed for the isolation of antibiotic-resistant bacteria, and isolates were characterized using an array of molecular and antibiotic susceptibility tests. Aerobic plate counts of the cooked samples (n = 13) varied from < 25 to 6.2 log CFU/g. Isolates obtained (n = 110) were spread across 18 genera, comprised of coliforms and opportunistic pathogens. Interestingly, isolates from cooked shrimp showed higher resistance towards chloramphenicol (18.6%) and tetracycline (20%), while those from raw shrimp exhibited low levels of resistance towards nalidixic acid (10%) and tetracycline (8.2%). Compared to wild-caught shrimp, the imported farm-raised shrimp harbored distinct gut microbiota communities and a higher prevalence of antibiotic-resistance genes in their gut. The presence of antibiotic-resistant strains in cooked shrimps calls for change in processing for their mitigation.

## Introduction

Antibiotic resistance is one of the severe threats faced by the world. In the U.S., more than 2.8 million people are infected with antibiotic-resistant strains each year, resulting in at least 35,000 deaths^1^. A review on global antibiotic resistance reported that by the year 2050, 10 million lives per year are at risk due to the rise of infection by antimicrobial-resistant strains^2^. According to the United States Department of Agriculture (USDA), ~ 97% of fish and shellfish consumed in the U.S. are imported from China, Ecuador, India, Indonesia, Thailand, and Vietnam^3,4^. In the large aquaculture operations, where farmed shrimps are cultivated as monoculture at high density, the prevalence of pathogens is one of the biggest challenges faced by the aquaculture industry^5^. Infection caused by pathogens in aquaculture operations can lead to massive production loss^6^ that can completely wipe out the shrimp farms^7^. The post-larvae infection caused by bacterial pathogens in shrimps in 2013 resulted in 1 billion dollars in production loss^6^. To prevent the production loss associated with bacterial infections, the aquaculture industry and shrimp hatcheries are heavily dependent on the application of antibiotics as prophylactic and therapeutic agents^8–12^. The use of multiple classes of antibiotics, e.g., tetracyclines (chlortetracycline, oxytetracycline, and tetracyclines), fluoroquinolone (enrofloxacin and ciprofloxacin), quinolones (oxolinic acid and norfloxacin), sulfonamides, chloramphenicol, and nitrofuran are practiced in shrimp farming^5,13,14^. Antibiotics are administered in aquaculture by mixing them in their diet or by adding them in rearing water. Continuous application of antibiotics by the shrimp hatcheries and farms facilitates the development of antibiotic-resistant bacterial (ARB) strains^15^. Non-biodegradable antibiotics with a long activity period apply selective pressures in an environment^16^. For instance, application of quinolones (oxolinic acid)^17^ in a high microbial load environment, forms a perfect combination for the selection of antibiotic-resistant strains in farm-raised shrimp. Once an antibiotic-resistance determinant has been selected, it can easily be transferred to other strains.

Popular foods like cooked shrimp or seafood mix are processed using mild heat treatment^18^ and can act as perfect vehicles for transferring ARB strains. Currently, FDA cooking recommendations are focused on mitigating *Listeria monocytogenes* in cooked shrimp^19^, which may not be effective to eliminate antibiotic-resistant bacteria spread over multiple genera. Further, antibiotic-resistant strains can be more tolerant to milder temperature treatments used for shrimp processing^20,21^. Cooked shrimp samples are mostly harvested and processed overseas. Each country and processing facility has its own processing specifications, due to which there is a considerable variation in the microbiological specifications of cooked shrimp available in the retail market. These samples that can be directly consumed after thawing or short heat treatment can be a perfect vehicle for ARB strains and opportunistic pathogens. Past studies have extensively studied the prevalence of antibiotic resistance in fresh produce^22^, beef^23^, poultry^24^, and manure from different livestock^25,26^. However, limited research has been conducted evaluating the prevalence of ARB strains in shrimp. Cross contact of raw shrimp with processed shrimp is another issue of concern^27^. The presence of ARB strains and the opportunistic pathogen can be detrimental to human health and limit treatment options.

Microbiome diversity has a significant impact on host health^28^. The competitive exclusion theory entails that the higher gut microbial diversity, the lower the possibility for pathogenic colonization^29^. The application of antibiotics at hatcheries and shrimp farms can cause dysbiosis. Researchers in the past have characterized and observed major gut microbiome composition differences among diseased and healthy shrimps^30^. Similarly, a comparison of the gut microbiome composition of farmed shrimp raised with antibiotics and wild-caught shrimp raised without antibiotics can be used for developing alternate sustainable methods for shrimp farming. Therefore, the aim of this study was to (a) isolate and characterize ARB strains among commercially available shrimp samples and (b) characterize the gut microbiome diversity of wild-caught shrimp from the U.S. compared to farm-raised shrimp imported from Ecuador.

## Results

### Distinct arrays of antibiotic resistance spectrum and the prevalence of antibiotic-resistant bacteria in raw versus cooked shrimp

A total of 31 shrimp samples (13 cooked and 18 raw shrimp) (Table 1) were tested for the presence of antibiotic-resistant bacteria. These shrimp samples originated from eight different countries i.e., Argentina (n = 2), Ecuador (n = 3), India (n = 6), Indonesia (n = 6), Panama (n = 1), Thailand (n = 3), U.S. (n = 7) and Vietnam (n = 3). Aerobic plate count (APC) and coliform count of cooked shrimp samples (n = 13) varied from < 25 CFU/g to 6.2 log CFU/g and < 25 CFU/g to 2.6 log CFU/g, respectively. All shrimp samples tested negative for *Salmonella*. Presumptive black colonies obtained on Xylose Lysine Deoxycholate agar with tergitol (XLD_tn_) and Hektoen Enteric (HE) agar plates tested negative using the *Salmonella* specific real-time PCR assay.

**Table 1:** Shrimp sample details for ABR study collected from Florida and Georgia.

Sample number	Sample detail	Processing state	Packaging state	Harvest method	Country of origin
1	Head on white shrimp	Raw	Loose	Wild caught	U.S
2	Head on pacific white shrimp	Raw	Loose	Farm raised	Ecuador
3	Head on pacific white shrimp	Raw	Loose	Farm raised	Ecuador
4	Head on white shrimp	Raw	Loose	Wild caught	U.S.
5	Head on pink shrimp	Raw	Loose	Wild caught	U.S.
6	Pacific white shrimp	Raw	Loose	Farm raised	Thailand
7	Raw shrimp	Raw	Sealed	Farm raised	Indonesia
8	Raw shrimp	Raw	Sealed	Farm raised	India
9	Extra small shrimp	Cooked	Sealed	Farm raised	Thailand
10	Small shrimp plater	Cooked	Sealed	Farm raised	Indonesia
11	Shrimp	Cooked	Sealed	Farm raised	Vietnam
12	Red shrimp	Raw	Sealed	Wild caught	Argentina
13	Shrimp	Raw	Sealed	Farm raised	Thailand
14	Jumbo shrimp	Cooked	Sealed	Farm raised	Indonesia
15	Extra-large shrimp	Cooked	Sealed	Farm raised	India
16	Medium shrimp	Cooked	Sealed	Farm raised	Indonesia
17	Jumbo white shrimp	Raw	Sealed	Wild caught	U.S.
18	Shrimp	Raw	Sealed	Farm raised	India
19	Shrimp	Cooked	Sealed	Farm raised	India
20	Shrimp	Cooked	Sealed	Farm raised	India
21	Shrimp	Cooked	Sealed	Farm raised	Indonesia
22	Shrimp	Cooked	Sealed	Farm raised	Indonesia
23	Shrimp	Raw	Loose	Farm raised	Ecuador
24	White shrimp	Raw	Loose	Wild caught	Panama
25	Royal red	Raw	Loose	Wild caught	Argentina
26	White shrimp	Raw	Loose	Wild caught	U.S.
27	White shrimp	Raw	Loose	Wild caught	U.S.
28	Wild gulf shrimp	Raw	Sealed	Wild caught	U.S.
29	Medium shrimp	Cooked	Sealed	Farm raised	Vietnam
30	Extra-large shrimp	Cooked	Sealed	Farm raised	India
31	Colossal shrimp	Cooked	Sealed	Farm raised	Vietnam

The shrimp samples (Table 1) were subsequently used for the isolation of extended-spectrum beta-lactam (ESBL) and carbapenem-resistant bacterial strains. After selective enrichment and plating on MacConkey agar media supplemented with specific antibiotics, a total of 120 bacterial isolates were obtained. Interestingly, no isolate was obtained from sample number 21 (cooked shrimp, farm-raised, Indonesia). Out of 120 isolates, 16S rRNA gene sequences were generated for 110 isolates following BLAST analysis. The 16S rRNA gene sequences generated were deposited to the NCBI nucleotide database and are available under NCBI GenBank accession numbers MT470929.1 to MT471033.1. The final 110 isolates represented 18 bacterial genera: *Acinetobacter* (n = 15), *Aeromonas* (n = 2), *Alcaligenes* (n = 1), *Citrobacter* (n = 18), *Escherichia* (n = 8), *Enterobacter* (n = 12), *Enterococcus* (n = 4), *Hafnia* (n = 1), *Klebsiella* (n = 2), *Lelliottia* (n = 5), *Morganella* (n = 14), *Obesumbacterium* (n = 2), *Pantoea* (n = 1), *Proteus* (n = 7), *Providencia* (n = 1), *Serratia* (n = 5), *Stenotrophomonas* (n = 3) and *Vibrio* (n = 9). The isolates obtained from cooked samples (n = 45) were dominated by *Morganella* (24%, n = 11), *Proteus* (11%, n = 5), *Lelliottia* (11.5%, n = 5), *Enterobacter* (9%, n = 4), *Enterococcus* (9%, n = 4) and *Citrobacter* (9%, n = 4) isolates. Whereas *Acinetobacter* (22%, n = 13), *Citrobacter* (23%, n = 13), *Enterobacter* (13%, n = 8), *Vibrio* (13%, n = 8), and *Escherichia* (10%, n = 6) were the top five genera isolated from raw shrimp samples.

**Figure 1:**
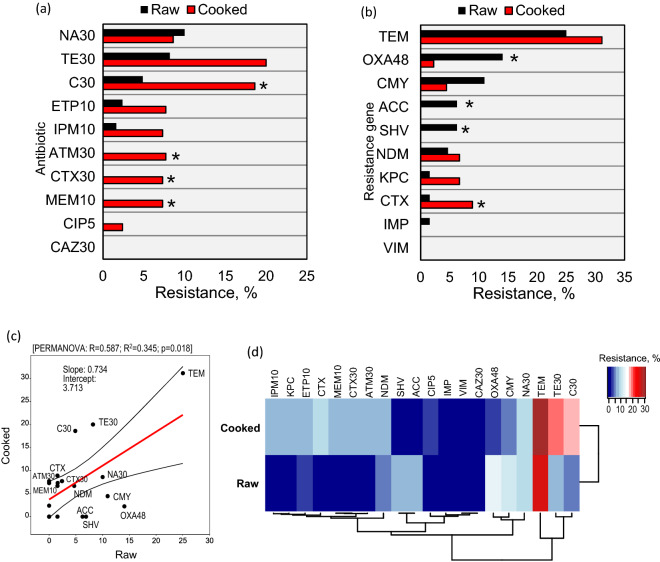
Differences in the prevalence of antibiotic-resistant bacteria in raw versus cooked shrimp. (**a**) Percentage resistance observed in isolates from cooked and raw shrimp samples by disc diffusion assay. (**b**) Real-time multiplex PCR assay for the detection of ESBLs and carbapenem genes. Isolates from cooked sample (n = 45) and raw samples (n = 62). (**c**) Generalized linear model demonstrating the profiles of antibiotic resistance percentage and the prevalence of resistance genes between raw versus cooked shrimp. (**d**) Heatmap clustering analysis showing the clusters of antibiotic resistance profiles and the resistant genes in raw versus cooked shrimp. Aztreonam (ATM-30), Cefotaxime (CTX-30), Ceftazidime (CAZ-30), Imipenem (IPM-10), Ertapenem (ETP-10), Meropenem (MEM-10), Tetracycline (Te-30), Ciprofloxacin (CIP-5), Nalidixic Acid (NA-30), Chloramphenicol (C-30). *p < 0.05.

Disc diffusion susceptibility assay was performed on 107 isolates. Three isolates [*Citrobacter freundii* (17B MM1)*, Morganella morganii* (20A MM1)*,* and *Vibrio parahaemolyticus* (7B MM)] were excluded from the study due to the lack of susceptibility standards. Data from the disc diffusion assay showed higher resistance towards tetracycline (9/45, 20%) and chloramphenicol (8/43, 19%) among isolates obtained from the cooked sample (Fig. 1a). Intermediate levels of resistance were observed among cooked sample isolates for ciprofloxacin, imipenem, and chloramphenicol at 17%, 12%, 7%, respectively. The most common resistance phenotype observed among cooked samples was tetracycline-chloramphenicol (C-TE). Interestingly, strains of *Stenotrophomonas maltophilia* were isolated from cooked, medium-sized shrimp samples imported from Vietnam. To our knowledge, this is the first report of the isolation of *S. maltophilia* from cooked shrimp samples.

Among isolates obtained from raw shrimp samples, lower levels of antibiotic-resistance were observed. Resistance towards tetracycline, nalidixic acid, chloramphenicol, and imipenem was observed at 8.1% (5/62), 10% (4/40), 5% (2/40), and 1.6% (1/62), respectively (Fig. 1a). However, high levels of intermediate resistance were observed towards cefotaxime (24.2%, n = 15/62), nalidixic acid (22.5%, n = 9/40), ciprofloxacin (11%, n = 6/53), and chloramphenicol (10%, n = 4/40). Surprisingly, all *Acinetobacter* isolates showed intermediate resistance towards cefotaxime. Raw shrimp isolates were found to be highly susceptible to ceftazidime, ciprofloxacin, meropenem, aztreonam, and ertapenem antibiotics by disc diffusion assay.

The minimum inhibitory concentration (MIC) data using the GN2F Sensititer Gram-Negative plates for six strains confirmed the presence of multiple drug resistance (MDR) strains in raw as well as cooked shrimp samples (Supplementary Table 1). The isolates from cooked shrimp (i.e.,* M. morganii* 31A MNC, *Proteus mirabilis* 20B-MC1 and *Serratia marcescens* 10A-MNC) showed resistance and intermediate resistance towards multiple antibiotics belonging to beta-lactams, cephalosporins, and nitrofurantoin class (Supplementary Table 1). However, the raw shrimp isolates *E. hormaechei* 2B-MC1, *S. marcescens* 28B-MC2, and *Vibrio parahaemolyticus* 24B-MC2 were resistant to 3 to 7 different antibiotics belonging to ampicillin, cephalosporin, fluoroquinolones, and nitrofurantoin. Further, whole-genome sequencing of isolate *E. hormaechei* 2B-MC1 showed the presence of *sul*1, *sul*2, *qnr*A1, *oqx*B, *dfr*A23, *bla*ACT, *flo*R, *fos*A, *tet*(A), *aph*(6)-Id, and *aph*(3″)-Ib resistance genes^31^. Based on the observed antibiotic profile, all isolates were classified as MDR isolates. The real-time PCR assay for the detection of ESBL resistance and carbapenem resistance genes showed a high prevalence of *bla*_TEM_ (31.1%), *bla*_OXA-48_ (9.2%), and *bla*_CMY_ (9.2%) genes among isolates (Fig. 1b). As shown in Fig. 1c, further analysis of the generalized linear model on the antibiotic susceptibility profiles and the prevalence of resistance genes in raw versus cooked shrimp also revealed a significant (p = 0.018) difference between the two groups. Figure 1d, in the form of hierarchical heatmap clustering analysis, also clearly demonstrates distinct signatures of antibiotics susceptibility array and the prevalence of resistance genes between the raw versus cooked shrimp.

### Distinct signatures of gut microbiome communities and antibiotic-resistant bacteria in wild-caught versus farm-raised shrimp

**Figure 2:**
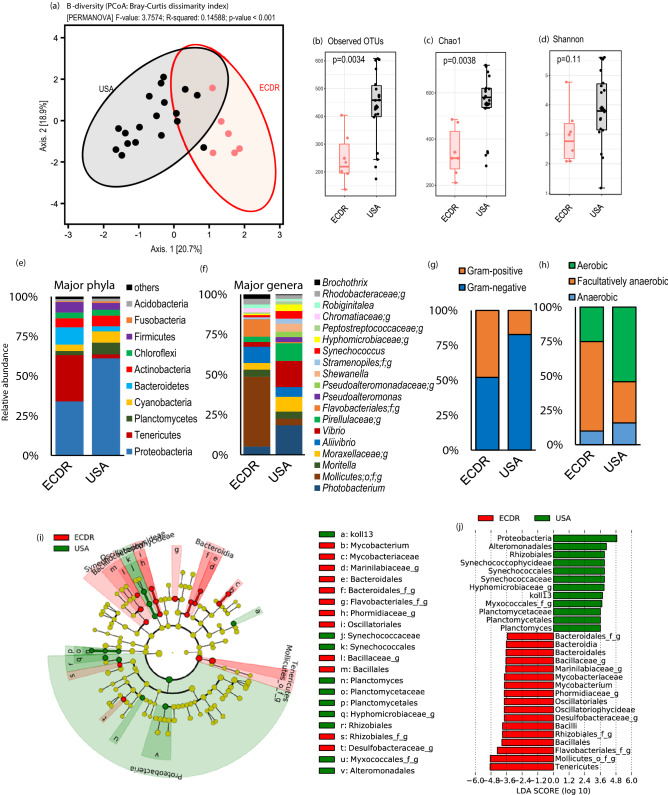
Distinct bacterial microbiome signatures in shrimp from U.S. (wild-caught) versus Ecuador (farm-raised) shrimps. (**a**) Beta-diversity (principal coordinate analysis; Bray–Curtis dissimilarity index; (**b–d**) alpha-diversity indices; (**e**,**f**) microbiome composition at phylum and genus level; (**g**,**h**) ratio of gram-positive to gram-negative bacteria and aerobic to anaerobic bacteria; and (**i**,**j**) linear discriminatory analysis effect size (LEfSe) cladogram representing the significantly unique bacterial taxa driving the difference between the wild-caught (U.S.) versus farm-raised (Ecuador) shrimp.

To further investigate whether and how the overall gut microbiome communities associated with these shrimps differ by geographical origin as well as the rearing method (farm vs. wild-caught), the 16S rRNA metagenomics analysis of the gut microbiome associated with the two groups of shrimps, i.e., farm-raised (from Ecuador) versus wild-caught (from the U.S.) was performed. Shrimp samples collected from countries with a smaller number of samples were removed from the analysis. Interestingly, the analysis revealed remarkably distinct spectrums of microbiome signatures between the two groups of shrimps (Fig. 2). The analysis of β-diversity (in terms of the principal coordinate analysis of the Bray–Curtis index) clearly demonstrated significantly discrete spectrums of microbiome signatures between shrimp from the U.S. versus those from Ecuador (Fig. 2a). Further α-diversity analyses in terms of observed operational taxonomic units (OTU), Chao1 index as well as the Shannon diversity index revealed higher bacterial species richness and diversity in wild-caught shrimps from the U.S. compared with shrimps from Ecuador (Fig. 2b–d). Accordingly, further analysis at the level of major bacterial phyla and genera showed clearly distinct microbiome composition between the two groups of shrimps (Fig. 2e–f). Compared to farm-raised shrimps from Ecuador, the wild-caught shrimps from the U.S. harbored a higher abundance of Proteobacteria and Planctomyces and had a lower abundance of Tenericutes and Bacteroidetes (Fig. 2e). Interestingly, the analysis of organism-level phenotypes showed that the shrimps from the U.S. harbor a significantly higher overall ratio of Gram-negative to Gram-positive bacterial taxa as compared to shrimps from Ecuador (Fig. 2g). In addition, the proportion of aerobic taxa was higher while that of facultative anaerobic taxa was lower in shrimps from the U.S. versus those from Ecuador (Fig. 2h). Linear discriminatory analysis (LDA) effect size (LEfSe) analysis was performed to infer the relative abundance of unique taxonomic clades and Kyoto Encyclopedia of Genes and Genomes (KEGG) metagenome orthologs that were significantly (p < 0.01) over-or under-represented between shrimp from Ecuador and the U.S.. The LEfSe analysis identified several unique bacterial taxa that drive differences in the gut communities between the two groups of shrimps (Fig. 2i). Figure 2j further simplifies these data and presents the LDA scores of the taxa that were significantly (p < 0.01) different between the two groups of shrimps. The shrimps from the U.S. were uniquely distinguished by a higher proportion of Proteobacteria*,* Alteromonadales, Rhizobiales, Synechococcaceae, Myxococcales, and Planctomyces, whereas shrimps from Ecuador were characterized by a higher abundance of Tenericutes*,* Flavobacteriales*, Desulfovibrio,* Oscillatoriales, *Mycobacterium,* and Bacteroidales (Fig. 2i–j). In addition, data from the study showed several bacterial species that were significantly different between the two groups of shrimps (Supplementary Fig. 1a). These differentially abundant markers could be considered as potential metagenomic biomarkers for the identification of shrimp from Ecuador (farm-raised) and the U.S. (wild-caught). Further, the PICRUSt (Phylogenetic Investigation of Communities by Reconstruction of Unobserved States) analysis of the KEGG orthologs associated with these bacterial signatures also revealed distinct clusters among the two groups (Supplementary Fig. 1b). The shrimps from the U.S. were characterized mainly by a higher (p < 0.01) abundance of bacterial taxa associated with the metabolism of several amino acids, lipids, cofactors, and vitamins in addition to the xenobiotic degradation (Supplementary Fig. 1b). In contrast, the shrimps from Ecuador presented a higher proportion of taxa associated with the metabolism of amino acid-related enzymes and gluconeogenesis in addition to the taxa related to primary immunodeficiency (Supplementary Fig. 1b).

**Figure 3:**
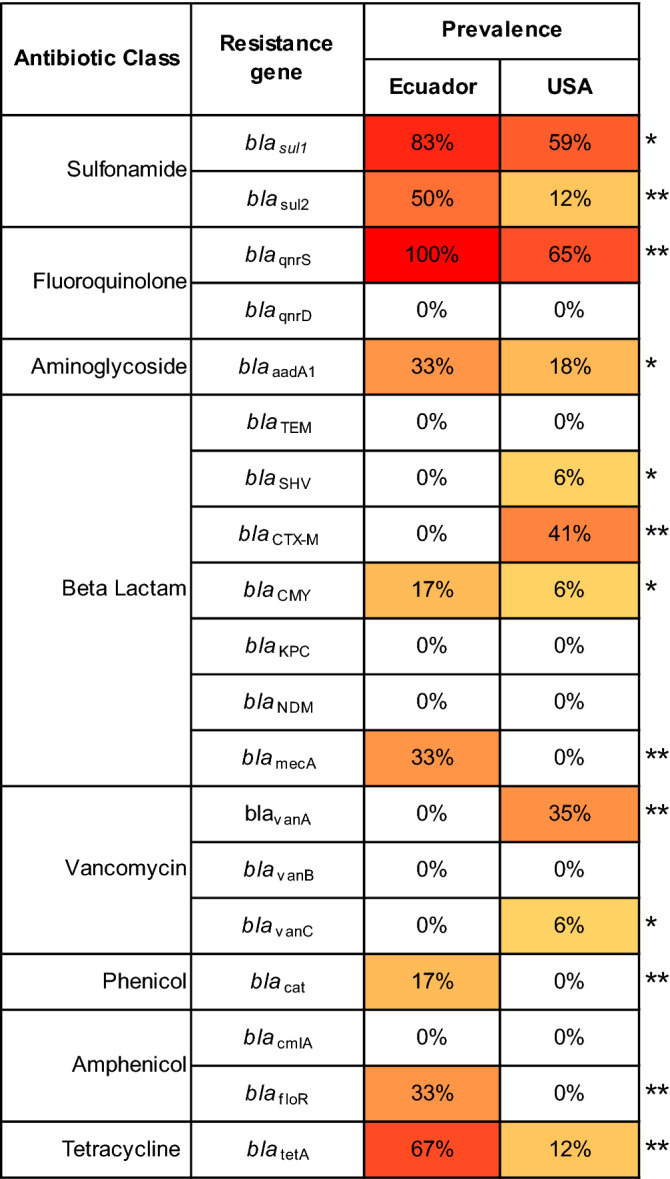
Differences in the prevalence of antibiotic-resistant bacteria in wild-caught (U.S.) versus farm-raised (Ecuador) shrimp. Percentage resistance observed among bacterial genomic DNA isolated from shrimp gut samples by real-time PCR assays. *p < 0.05; **p < 0.01.

In addition to gut microbiome analysis, the bacterial genomic DNA from raw shrimp gut from the U.S. (wild-caught) and Ecuador (farm-raised) was subjected to real-time PCR assays for examining the prevalence of antibiotic-resistance genes. Interestingly, compared to raw shrimp sample of U.S. origin (wild-caught), the shrimp from Ecuador (farm-raised) showed a significantly higher prevalence of *bla*_sul-1_, *bla*_sul-2_, *bla*_qnrS_, *bla*_aadA_, *bla*_mec-A_, *bla*_CMY_, *bla*_floR_, *bla*_cat_, and *bla*_tet-A_ (p < 0.01) while demonstrating a lower prevalence of *bla*_CTX-M_ and *bla*_van-A_ (p < 0.01) (Fig. 3).

## Discussion

According to the Food and Agricultural Organization (FAO), the microbiological limits for frozen cooked shrimp samples with aerobic plate count 10^7^ CFU/g is the boundary between marginally acceptable counts and unacceptable counts^32^. In our study, the APC of cooked shrimp samples ranged from < 25 CFU/g to 6.2 logs CFU/g. Cooked shrimp samples from India (sample No. = 30) and Vietnam (sample No. = 31) had a high APC. Variations in  hazard analysis critical control point and processing plans in different countries and temperature fluctuation during transportation may be possible reasons for the observed variation. Some samples showed as low as < 25 CFU/g APC, while other samples had high APC and even tested positive for the presence of antibiotic-resistant bacteria. We found that 22% (n = 24/107) of the bacterial isolates were resistant to one or more antibiotics. Interestingly, compared to isolates from raw shrimp, the resistance among isolates from cooked shrimp was significantly higher towards chloramphenicol (19%), aztreonam (7.7%), cefotaxime (7.3%), and meropenem (7.3%) (p < 0.01) (Fig. 1a). In a similar study, Nawaz et al.^33^ reported resistance to nalidixic acid, ampicillin, tetracycline, and chloramphenicol in 52% (55/105) of *E. coli* isolates from imported black Asian shrimp (n = 330).

Shrimp processing facilities rely on the application of a shorter and milder thermal treatment, which may not be sufficient for mitigation of ARB strains spread over multiple genera. Several studies have demonstrated the interplay between stress associated with food processing, antibiotic resistance, and vice-versa^34^. In fact, exposure to mild temperatures has been shown to enhance the selection of ARB strains. Ebinesh et al. observed a reduction in the inhibition zone for amikacin, imipenem, meropenem, norfloxacin, piperacillin, and tazobactam among six *A. baumannii* strains following heat treatment at 45 °C^21^. In another study, the ARB strains of *E. coli* O157:H7 were found to be less susceptible to 55 °C treatment compared to the wild strain^20^. Hwang et al. reported survival of antibiotic-resistant strains of *E. coli* with the *tet*(A) gene in the human gastrointestinal tract after consumption of retail ready-to-eat food contaminated ARB *E. coli* strains^35^. They further reported the transfer of the resistance gene from the ARB strain associated with food to antibiotic sensitive cells in the human gastrointestinal tract^35^. Commercially available cooked shrimp samples with high APC are not likely to cause infection in healthy individuals. However, the consumption of frozen shrimp harboring antibiotic-resistant strains of opportunistic pathogens can lead to serious illnesses, particularly in immunocompromised populations. The presence of ARB strains in cooked food may promote the transfer of resistance genes to bacterial strains in the human gut microbiome or pathogens.

Oxytetracycline is widely used by hatcheries across the world^36,37^. Along with the widespread use of oxytetracycline for aquaculture, resistance to this drug is commonly observed in bacterial isolates from shrimp^38^. In the present study, tetracycline resistance was associated with *Enterobacter hormaechei*, *Enterococcus faecalis*, *Morganella morganii*, *Proteus mirabilis,* and *Vibrio parahaemolyticus* isolates from the shrimp imported from India, Vietnam, and Indonesia. Chloramphenicol, a broad-spectrum antibiotic, is banned for use in food-producing animals by the U.S., European Union, and many other countries due to its toxic side-effects in humans^39^. However, the presence of chloramphenicol residue is a common reason for the rejection of imported shrimp by the FDA^40^. The presence of chloramphenicol residues reflects the indiscriminate use of banned antibiotics in shrimp-exporting counties. In the present study, chloramphenicol resistance was associated with *Enterobacter hormaechei*, *Enterobacter lignolyticus*, *Proteus mirabilis*, *Morganella morganii*, and *Serratia marcescens* isolates. In cooked samples, chloramphenicol resistance was associated with *Enterococcus faecalis, M. morganii, P. mirabilis,* and *S. maltophilia*, all of which are known to be opportunistic nosocomial pathogens and cause infection in the immunosuppressed population^41^. Not many reports are available on the isolation of carbapenem-resistant bacteria from food-producing animals, including shrimp and their environment^42^. A pilot study conducted by the National Antimicrobial Resistance Monitoring System (NARMS) reported carbapenem-resistant isolates with *bla*_NDM-1_ resistance genes in *Acinetobacter baumannii* and *Aeromonas sobria* from seafood samples collected from the U.S.^43^. In our study, carbapenem-resistance was observed in strains belonging to *E*. *hormaechei*, *P*. *mirabilis*, and *S*. *maltophilia*. Additionally, a small percentage of isolates from both raw and cooked shrimp samples tested positive for the presence of *bla*_KPC_, *bla*_NDM_, *bla*_OXA48,_ and *bla*_IMP_ carbapenem-resistance genes. These genes were associated with *Acinetobacter pittii* (*bla*_OXA48_), *Citrobacter* spp. (*bla*_OXA48_), *C. freundii* (*bla*_KPC_ and *bla*_OXA48_), *Enterobacter cloacae* (*bla*_KPC_ and *bla*_NDM_), *E*. *hormaechei* (*bla*_NDM_ and *bla*_OXA48_), *E. lignolyticus* (*bla*_OXA48_), *Klebsiella pneumoniae* (*bla*_OXA48_), *M*. *morganii* (*bla*_OXA48_), *Vibrio fluvialis* (*bla*_IMP_), and *V*. *parahaemolyticus* (*bla*_OXA48_). The carbapenem-resistance genes are plasmid-borne, which can facilitate their spread in the food-producing environment and can promote the development of co-resistance^42^.

In the present study, low levels of resistance were observed towards ESBL antibiotics. All isolates were susceptible to ceftazidime, 7.7% of isolates showed resistance to cefotaxime and aztreonam, and 16.6% of isolates showed intermediate resistance to cefotaxime. Intermediate resistance to cefotaxime was primarily associated with strains belonging to the *Acinetobacter pittii*. A previous study also reported resistance to third-generation (cefotaxime, ceftriaxone, and ceftazidime) and fourth-generation (cefepime) cephalosporins in two *E. cloacae* isolate from fresh seafood^44^*.* A similar study reported the isolation of ESBL-producing Enterobacteriaceae from fresh seafood samples collected from the India market, wherein 80% (n = 53/66) of *E. coli* isolates were found to be ESBL producers^45^. MDR phenotype was reported among 169 isolates with resistance to third-generation cephalosporins, aztreonam, ertapenem, and meropenem^45^. Further, the isolates from the study were found to be positive for ESBL-encoding genes *bla*_CTX_, *bla*_SHV,_ and *bla*_TEM_ and the metallo-β-lactamase gene *bla*_NDM-1_. The extensive use of unapproved antibiotics for aquaculture operations in India can be a possible reason for this higher prevalence of antibiotic-resistant bacteria in shrimp harvested from India. In 2019, a total of 61 shrimp entry lines were refused by the FDA due to the presence of banned antibiotic residues from different countries. Among all refused products, shrimp products exported from India (n = 33/61) have consistently tested positive for banned antibiotics reflecting extensive use of banned antibiotics for aquaculture in India^46–48^.

The strains of *S. maltophilia* were isolated from farm-raised shrimp from Vietnam and following the disc diffusion assay antimicrobial susceptibility testing (AST) analysis of the strain confirmed its intrinsic resistance (i.e., towards chloramphenicol and ceftazidime)^49^. Vietnam allows the use of 32 antibiotics for aquaculture^50^. Due to the extensive use of a wide range of antibiotics in Vietnam, 2-hydroxy-3 phenylpyrazine (HPP), ampicillin, and β-lactam antibiotic degraded products were detected in 60 water samples collected from rivers (11/26) and household ponds (49/62)^51^. Yamasaki et al.^52^ reported ciprofloxacin (4/247), enrofloxacin (12/247), oxolinic acid (1/247), sulfamethazine (2/247), sulfamethoxazole (2/247), and trimethoprim (1/247) residues in shrimp samples collected from Vietnam. Indiscriminate use of a wide range of antibiotics for aquaculture operation and contamination of aquaculture ponds by untreated hospital waste can be one of the possible reasons for the presence of MDR strains of *M. morganii* and *S. maltophilia* isolated in our study.

Based on the observed antibiotic resistance profile by disc diffusion assay, we selected six isolates and further characterized their antibiotic-susceptibility array using Sensititer Gram-Negative GN2F plates. The data revealed resistance to beta-lactams, cephalosporins, quinolones, dihydrofolate reductase inhibitor/sulfonamide, and fluoroquinolones class of antibiotics and intermediate resistance to carbapenem antibiotic (i.e., imipenem) (Supplementary Table 1). Based on the updated definition of MDR viz. "the isolate is non-susceptible to at least 1 agent in ≥ 3 antimicrobial categories"^53^, all of the isolates tested herein using Sensititer Gram-Negative GN2F plates were characterized as MDR isolates. The widespread trade of shrimp can act as a vehicle for the dissemination of antibiotic-resistant genes. Currently, the FDA relies on only testing antibiotic residue^54^ in imported seafood. Hence, the presence of MDR strains in cooked shrimp samples points toward a gap in monitoring measures.

Despite emerging interest and research on marine and seafood-related microbiome, the microbial communities associated with shrimp gut remain relatively underexplored. In our study, the gut microbiota of shrimp samples collected from the U.S. had a higher abundance of Proteobacteria and Planctomyces and had a lower abundance of Tenericutes and Bacteroidetes. A study characterizing the gut microbiota of adult *Litopenaeus vannamei* raised in farms located in China reported that the adult shrimp gut from farm-A was dominated by Proteobacteria, Tenericutes, Firmicutes, Planctomyces, and Verrucomicrobia^55^. Parallel findings were reported wherein *L. vannamei* samples were collected from Mexico, showing a higher abundance of Proteobacteria, Cyanobacteria, Actinobacteria, Gemmatimonadetes, Bacteroidetes, and Firmicutes^56^.

Further, we observed higher bacterial species richness and diversity in wild-caught shrimps from the U.S. when compared to farm-raised shrimps from Ecuador. Rungrassamee et al. reported the presence of higher bacterial diversity in wild-caught *Penaeus monodon* (Tiger shrimp) compared to farmed-raised samples^57^. The major factor affecting the composition of aquatic organism gut microbiota are egg microbiota^58^, larval rearing water^59^, shrimp diet^60–62^, and environmental factors^63^. Fish on an herbivorous diet has been shown to have higher diversity compared to fish on a carnivorous diet^60–62,64^. Farm-raised shrimp are commonly fed an artificially formulated diet consisting of various animal proteins. A similar significant difference (p < 0.05) in the diversity indices were observed among the wild-caught and farm-raised *L. vannamei* samples collected from Mexico^56^, wherein Actinobacteria and Nitrospirae were found to be significantly abundant in wild-caught *L. vannamei* samples compared to Bacteroidetes, Gemmatimonadetes, Fusobacteria and Spirochaetes phyla, which were abundant in farm-raised samples^56^.

The two-dimensional PCoA plot for the beta-diversity for the shrimp samples from the U.S. and Ecuador showed clear clustering of representative samples into two distinct groups. Shrimp samples collected in our study were comprised of *Pandalus borealis* (Pink shrimp) and *Litopenaeus setiferus* (White shrimp). These two shrimp species clustered together in the PCoA plot, showing that the region of shrimp origin is a more crucial factor affecting gut microbiota than host genetics. Tzeng et al.^65^ characterized the gut microbiome composition of two closely related shrimp species, i.e., *Macrobrachium nipponense* (oriental river prawn) and *M. asperulum*, harvested from river and lake water located in Taiwan. *Penaeus monodon* samples were used as control. Data from the study showed that the host genetics and habitat both play important roles in determining the gut microbial composition of shrimp. Further, the PCoA plot from the study revealed that the host habitat has a much greater impact on the shrimp gut microbial composition. The effect of habitat on the gut microbial composition can be attributed to the similar diet and exposure to similar aquatic microbiota among different species of shrimp from a geographical region.

The PICRUSt data from a previous study showed an abundance of pathways dedicated to amino acid metabolism, lipid metabolism, and xenobiotics biodegradation in wild-caught *L. vannamei*^56^, which is similar to the PICRUSt data for wild-caught shrimp collected from the U.S. in our study. These distinct microbiome signatures observed among the U.S. wild-caught shrimp, when confirmed by further studies, could be used for designing region-specific probiotic therapies for improving shrimp health and can support the aquaculture of shrimp in the U.S. Pure culture strains of *E. hormaechei* were isolated in this study, which has been used in the past as a shrimp probiotic^66^.

In the present study, the prevalence of sulfonamide (*bla*_sul1_ and *bla*_sul2_), quinolone (*bla*_qnrS_), aminoglycoside (*bla*_aadA_), beta-lactam (*bla*_mecA_ and *bla*_CMY_), phenicol (*bla*_floR_ and *bla*_cat_), and tetracycline (*bla*_tetA_) resistance genes was significantly higher in the gut of shrimp imported from Ecuador (Fig. 3). The higher abundance of these antibiotic-resistance genes in farm-raised shrimp can be attributed to the application of antibiotics for aquaculture operations facilitating the selection of strains with resistance genes. The presence of these genetic elements can facilitate the transfer of selected genes to genetically related and unrelated strains via the food chain^67^. A previous study has also shown a strong positive correlation of the presence of *bla*_sul1_ and *bla*_qnrD_ genes with the abundance of Proteobacteria; *bla*_cmlA_ and *bla*_floR_ genes with the abundance of Bacteroidetes; *bla*_floR_ and *bla*_sul2_ with Firmicutes; and *bla*_cmlA_, *bla*_tetG_, *bla*_qnrB,_ and *bla*_qnrA_ with the abundance of Acidobacteria and Cyanobacteria^55^. Notably, these five phyla (i.e., Proteobacteria, Bacteroidetes, Firmicutes, Acidobacteria, and Cyanobacteria) were dominant phyla among farm-raised shrimp from Ecuador^55^.

One of the limitations of this study was our dependence on Sanger sequencing of 16S rDNA region for the identification of the obtained isolates. 16S rDNA sequencing is not very accurate for the differentiation of closely related species. To our knowledge, this is the first report demonstrating the isolation and prevalence of MDR strains of *S. maltophilia* from imported cooked shrimp. Selected cooked shrimp showed high APC as well as coliform counts. The data demonstrate that the presence of MDR bacterial strains and opportunistic pathogens in imported cooked shrimp products may pose a threat to human health and calls for the need for standardization of effective cooking time–temperature combination for potential mitigation of antibiotic-resistant strains in cooked shrimp samples. Further, data from our study can be used as baseline data for the U.S. wild-caught shrimp microbiome composition, and with larger validation studies, the microbiome markers identified in our study can be used for the development of novel PCR-based detection assays.

## Methods

### Shrimp samples

Raw (n = 18) and cooked (n = 13) shrimp samples were purchased from March to September 2019 from stores, supermarkets, and retailers located in the states of Florida and Georgia. The collected shrimp samples were either frozen pre-packaged or thawed shrimp placed on ice in bulk. The pre-packaged samples had a clear country of origin label. Similarly, thawed shrimp placed on ice in bulk collected from the supermarket had a country-of-origin label. Cooked shrimp samples were already peeled, deveined, and pre-cooked, and could be directly consumed after thawing. Sample details are listed in Table 1.

### Aerobic plate count and coliform count

Twenty-five grams of cooked shrimp samples from each collected sample were weighed in Whirl–Pak filtered stomacher bags (Nasco, MI, U.S.), and samples were diluted using 225 mL of sterile 0.1% peptone water (w/v). Samples were stomached at 230 rpm for 2 min and were serially diluted using 9 mL of 0.1% (w/v) peptone water tubes. Diluted samples were plated on plate count agar (Hardy Diagnostics, CA, U.S.) and violet red bile agar (Hardy Diagnostics, CA, U.S.). Plates were incubated at 37 °C for 48 h, and colonies were enumerated after the incubation period, and obtained counts were expressed in log10 CFU/mL.

### Isolation of *Salmonella*

Twenty-thirty grams of all shrimp samples were individually weighed in Whirl–Pak stomacher bags (Nasco, MI, U.S.). Samples were hand crushed and enriched with lactose broth (1:9) (Hardy Diagnostics, CA, U.S.) at 37 °C for 24 h. One mL and 0.1 mL of each enriched sample were transferred to freshly prepared 10 mL tetrathionate broth (T.T; Hardy Diagnostics, CA, U.S.) and Rappaport–Vassiliadis (R.V; Hardy Diagnostics, CA, U.S.) broths for selective enrichment, respectively. Inoculated samples in R.V. and T.T. tubes were incubated for 24 h at 42 ± 1 °C and 35 ± 1 °C, respectively. Samples after selective enrichment were streaked onto an XLD plate (Hardy Diagnostics, CA, U.S.) containing 4.6 mL/L tergitol (Niaproof; Sigma, MO, U.S.) and 15 mg/L of novobiocin (Alfa Aesar, MA, U.S.) and HE agar plates. After a 24 h incubation period, plates were observed for black colonies. DNA from black colonies was isolated using PrepMan Ultra Sample Preparation reagent (Applied Biosystems, CA, U.S.) and used for real-time PCR assay for specific detection of *Salmonella*^68^.

### Isolation of ESBL and carbapenem-resistant bacterial strains

Collected shrimp samples were screened for the presence of ESBL resistant isolates as previously described. Briefly, 20–30 g of shrimp sample were transferred to Whirl–Pak stomacher bags (Nasco, MI, U.S.). As the exoskeleton of shrimp can rupture the stomacher bags, samples were hand crushed by applying pressure on the stomacher bags. Each shrimp sample was diluted with 225 mL of phosphate-buffered tryptic soy broth (TSB) with 2 µg/mL cefotaxime. After 18 h incubation, samples were streaked on following agars: (a) MacConkey agar with 4 µg/mL cefepime; (b) MacConkey agar with 4 µg/mL cefoxitin; (c) MacConkey agar with 8 µg/mL cefoxitin, 4 µg/mL cefepime, and 0.5 µg/mL meropenem. Two colonies with distinct colony morphology were selected from each plate and streaked on their respective plates for further purification. Isolated colonies were sub-cultured in TSB and preserved as a 20% (v/v) glycerol stock and stored in a − 20 °C freezer.

### Molecular characterization of bacterial isolates

DNA was isolated from overnight broth cultures using PrepMan Ultra Sample Preparation reagent (Applied Biosystems, CA, U.S.). The quality and quantity of isolated DNA were measured using a NanoDrop One Spectrophotometer (Thermo Fisher Scientific, MA, U.S.), and samples were diluted to 20 ng/µL. The 16S rDNA sequences were amplified using 27F: AGAGTTTGATCMTGGCTCAG and 511R: GCGGCTGCTGGCACRKAGT primer pair^69^. Sanger sequencing of resulting amplicons were performed at the DNA Sequencing Facility of the Florida State University (Tallahassee, Florida). Sequence data were BLAST analyzed for the confirmation of genus and species identification of isolates.

### Antibiotic susceptibility testing

The AST of all obtained isolates were performed using the disk diffusion method according to Clinical and Laboratory Standards Institute (CLSI) guidelines with *Escherichia coli* ATCC 25922 and *Staphylococcus aureus* ATCC 25923 as quality control strains. Enterobacteriaceae strains were tested for resistance to tetracycline (TE), beta-lactam antibiotics [aztreonam (ATM-30)], cephalosporins [cefotaxime (CTX-30), ceftazidime (CAZ-30)], carbapenem antibiotics [imipenem (IMP-10), ertapenem (ETP-10), meropenem (MEM-10)], fluoroquinolones [ciprofloxacin (CIP-5) and nalidixic acid (NA-30)] and phenicols [chloramphenicol (C-30)] (Hardy Diagnostics, CA, U.S.). *Acinetobacter* isolates were tested against IMP, CAZ, CTX, CIP, T.E., and MEM; *Enterococcus* isolates were tested against C, CIP, and T.E.; *Stenotrophomonas* isolates were tested against CAZ and C, and resistance of *Vibrio* isolates were tested using IMP, CAZ, CTX, T.E., and MEM antibiotic discs. Data obtained from the disc diffusion assay were used to categorize the strains as susceptible, intermediate, or resistant based on CLSI guidelines (document; M100-S23, M100-S24, and M100-S26)^49,70,71^.

The MIC of six selected strains was determined using GN2F Sensititer Gram-Negative plates (TREK Diagnostic Systems, OH, U.S.) as per manufacturer instructions. *E. coli* ATCC 25922 and *S. aureus* ATCC 25923 were used as quality control strains. The six isolates were selected based on MDR profiles towards different antibiotic classes observed by the disc diffusion assay, i.e.,* Enterobacter hormaechei* 2B-MC1, *Morganella morganii* 31A-MNC, *Proteus mirabilis* 20B-MC1-1, *Serratia marcescens* 10A-MNC, *Serratia marcescens* 28B-MC2, and *Vibrio parahaemolyticus* 24B-MC2*.* The MIC data were analyzed based on CLSI guidelines (document M100-S29)^72^ and M45-02, 2010 edition for *Vibrio parahaemolyticus*^73^.

### Detection of antibiotic-resistance genes using real-time PCR assays

Pure culture bacterial DNA isolated from broth culture was used for real-time PCR assays. DNA samples were tested for the presence of antibiotic resistance genes (*bla*_KPC_, *bla*_OXA-48_, *bla*_NDM_, *bla*_VIM_, *bla*_CTX-M_, *bla*_IMP_, *bla*_CMY_, *bla*_ACC_, *bla*_SHV_, and *bla*_TEM_) by real-time PCR assay as previously described^55,68,74,75^.

### Microbiome analysis

Shrimp gut microbiome was analyzed according to our previously described methods^76–78^. In brief, the Earth Microbiome Project (EMP) benchmarked protocol^79^ was used for the characterization of shrimp gut microbiome. Microbial community analysis, protocols, and standards^80^ were employed by following a barcoded high-throughput sequencing approach, as described by Caporaso et al.^81^. Bacterial genomic DNA from the gastrointestinal tract of raw shrimp samples (Table 2) from the U.S. and Ecuador was extracted using the PowerFecal DNA kit (Qiagen, CA, U.S.). The V4 hypervariable region of the 16S rDNA gene was PCR-amplified using the universal primer pair 515F/806R^81^; the resulting uniquely barcoded amplicons were purified using Agencourt AMPure XP magnetic purification beads (Beckman Coulter, CA, U.S.) and quantified with Qubit fluorimeter (Qubit3; Invitrogen, CA, U.S.) and the dsDNA H.S. assay kit (Life Technologies, CA, U.S.); and the amplicon library was generated according to methods of Caporaso et al.^81^. The purified PCR products were pooled in equal molar concentrations and sequenced on one 2 × 300-bp Illumina MiSeq run (Miseq reagent kit v3; Illumina Inc., CA, U.S.) for paired-end sequencing. The sequencing quality control was carried out with on-board Miseq Control Software and Miseq Reporter (Illumina Inc.). The resultant sequences were de-multiplexed, quality-filtered, clustered and taxonomically-assigned (at 97% similarity level against GreenGenes database, May 2013 version) with Ribosomal Database Project (RDP)-classifier workflow as described by Wang et al.^82^ using QIIME software suite^83^ as described previously^76,78^. Quality filtering was performed as described previously^84^. Subsequent data processing included filtering by clustering similar sequences with less than 3% dissimilarity by using the USEARCH algorithm version 5.2.32^85^ with an open-reference OTU clustering method using the Greengenes database and detecting and removing chimeras by using the UCHIME algorithm^86^. The most abundant sequence in each OTU was selected as the representative sequence and the resulting OTUs were assigned to taxa by using the Ribosomal Database Project classifier^82^ trained on the Greengenes reference database. A total of 2,183,494 sequences (mean ± SEM: 77,981.929 ± 7707.829) were obtained after quality-filtering. To avoid the bias of sequencing depth, the sequences were rarefied to the minimum sequence reads per sample for downstream analyses. To avoid the bias of sequencing errors or low-level contaminations, the OTUs with very low read count (less than 4) in very few samples (less than 10% prevalence) were filtered out from the subsequent analyses. The data of taxon abundance were subjected to the total sum scaling, and the taxa with less than 1% mean relative abundance were further excluded from the subsequent downstream analyses. To avoid the bias of nucleic acid extraction, PCR reaction conditions, and primers on community composition obtained by amplicon sequencing, all the samples were batch-processed identically and simultaneously. Bacterial community composition was measured at taxonomic levels of phylum, class, order, family, and genus. Alpha-diversity measures were calculated within QIIME. Beta-diversity was computed using principal coordinate analysis (PCoA) of the Bray–Curtis dissimilarity index, as described previously^87^. The functional activities of the bacterial communities were analyzed using the open-source bioinformatics tool PICRUSt (Phylogenetic Investigation of Communities by Reconstruction of Unobserved States; http://picrust.github.io/picrust/index.html) as described previously^88^, wherein the sequencing dataset was normalized and the metagenomes were predicted against the PICRUSt-formatted, characterized-protein, functional database of Kyoto Encyclopedia of Genes and Genomes (KEGG www.kegg.jp/kegg/kegg1.html)^89^.

**Table 2:** Shrimp sample details for microbiome study collected from supermarkets located in Florida and Georgia.

Sample number	Sample detail	Scientific name	Cultivation method	Country of origin
1	White shrimp	*Litopenaeus setiferus*	Wild caught	U.S.
2	Pacific white shrimp	*Litopenaeus vannamei*	Farm raised	Ecuador
3	Pacific white shrimp	*Litopenaeus vannamei*	Farm raised	Ecuador
4	White shrimp	*Litopenaeus setiferus*	Wild caught	U.S.
5	Head on pink shrimp	*Pandalus borealis*	Wild caught	U.S.
17	White shrimp	*Litopenaeus setiferus*	Wild caught	U.S.
23	Pacific white shrimp	*Litopenaeus vannamei*	Farm raised	Ecuador
26	White shrimp	*Litopenaeus setiferus*	Wild caught	U.S.
27	White shrimp	*Litopenaeus setiferus*	Wild caught	U.S.
34	Pink shrimp	*Pandalus borealis*	Wild caught	U.S.
35	Hopper pink shrimp	*Pandalus borealis*	Wild caught	U.S.
36	Hopper pink shrimp	*Pandalus borealis*	Wild caught	U.S.
37	Hopper pink shrimp	*Pandalus borealis*	Wild caught	U.S.
38	Hopper pink shrimp	*Pandalus borealis*	Wild caught	U.S.
39	White shrimp	*Litopenaeus setiferus*	Wild caught	U.S.
40	White shrimp	*Litopenaeus setiferus*	Wild caught	U.S.
41	White shrimp	*Litopenaeus setiferus*	Wild caught	U.S.
42	White shrimp	*Litopenaeus setiferus*	Wild caught	U.S.
43	White shrimp	*Litopenaeus setiferus*	Wild caught	U.S.
44	White shrimp	*Litopenaeus setiferus*	Wild caught	U.S.
45	White shrimp	*Litopenaeus setiferus*	Wild caught	U.S.
46	Pacific white shrimp	*Litopenaeus vannamei*	Farm raised	Ecuador
47	Pacific white shrimp	*Litopenaeus vannamei*	Farm raised	Ecuador
48	Pacific white shrimp	*Litopenaeus vannamei*	Farm raised	Ecuador

### Prevalence of antibiotic resistance genes in raw shrimp gut

Prevalence of antibiotic resistance genes in shrimp gut was determined using DNA isolated from shrimp gut and real-time PCR assays. Presence of antibiotic resistance gene (i.e., *bla*_sul-1_, *bla*_sul-2_, *bla*_qnrS_, *bla*_qnrD_, *bla*_aadA_, *bla*_TEM_, *bla*_SHV_, *bla*_CTX-M_, *bla*_CMY_, *bla*_KPC_, *bla*_NDM_, *bla*_mecA_, *bla*_vanA_, *bla*_vanB_, *bla*_vanC_, *bla*_cat_, *bla*_cmlA_, *bla*_floR_, and *bla*_tetA_) were tested as using primer-pair and PCR conditions as previously described^55,68,75,90^.

### Data analysis

Alpha-diversity indices and bacterial abundance data between the two groups of shrimps were compared by two-tailed unpaired t-test. The data of the prevalence of antibiotic resistance and the associated genes were compared with Fisher's exact test. LEfSe (Linear discriminatory analysis [LDA] Effect Size) was applied to identify the bacterial taxa driving the differences between the different groups of shrimps^91^, wherein the alpha parameter significance threshold for the Kruskal–Wallis as well as the Wilcoxon test implemented among classes was set to 0.01, and the logarithmic LDA score cut-off was set to 3.0. Bray–Curtis similarity scores inferred from the taxonomic data were reduced to a two-dimensional space using principal coordinate analysis (PCoA) for the computation of structural similarity (beta-diversity) of bacteriomes from different groups of shrimps. Differences in beta-diversity were computed by permutational multivariate analysis of variance (PERMANOVA) using the algorithm of web-based tool Microbiome Analyst^92^. Hierarchical clustering heatmaps were constructed within 'R' statistical software package (version 3.6.1; https://www.r-project.org/) using the 'heatmap.2’ and "ggplots" packages. Unless otherwise stated, the data are presented as means ± SEM. P < 0.05 was considered statistically significant unless specified.

## Supplementary Information

Supplementary Information.

## Data Availability

All data generated or analyzed during this study are included in this published article (and its Supplementary Information files). The raw sequences have been deposited at the Sequence Read Archive (SRA) under the SRA number (SUB7840299) and BioProject number PRJNA648917 for unrestricted, public access. Other information/ data related to the current study are available from the corresponding author on reasonable request.

## Abbreviations

APC: Aerobic plate count
ATM-30: Aztreonam
C-30: Chloramphenicol
CAZ-30: Ceftazidime
CFU: Colony-forming unit
CIP-5: Ciprofloxacin
CTX-30: Cefotaxime
EMP: Earth microbiome project
ESBL: Extended-spectrum β-lactam
ETP-10: Ertapenem
HE: Hektoen enteric
IMP-10: Imipenem
KEGG: Kyoto encyclopedia of genes and genomes
LDA: Linear discriminatory analysis
LEfSe: Linear discriminatory analysis effect size
MEM-10: Meropenem
NA-30: Nalidixic acid
PCoA: Principal coordinate analysis
PICRUSt: Phylogenetic Investigation of Communities by Reconstruction of Unobserved States
R.V.: Rappaport–Vassiliadis
T.E.: Tetracycline
TSB: Tryptic soy broth
T.T.: Tetrathionate broth
XLDtn: Xylose lysine deoxycholate agar with tergitol
