# Supplementary material for: Antibiotic-resistant bacteria and gut microbiome communities associated with wild-caught shrimp from the United States versus imported farm-raised retail shrimp
Source: Sci Rep. 2021 Feb 8;11:3356. doi: 10.1038/s41598-021-82823-y (PMC7870836; doi:10.1038/s41598-021-82823-y)
Supplement: Supplementary file 1 — Supplementary Information. [file 41598_2021_82823_MOESM1_ESM.docx]

Research Article

**Antibiotic-resistant bacteria and gut microbiome communities associated with wild-caught shrimp from United States versus imported farm-raised retail shrimp**

Laxmi Sharma^1^, Ravinder Nagpal^2^, Charlene R. Jackson^3^, Dhruv Patel^4^, Prashant Singh^1^*

^1^Department of Nutrition, Food and Exercise Sciences, Florida State University, Tallahassee, Florida, USA.

^2^Department of Microbiology and Immunology, Center for Diabetes, Obesity & Metabolism, Wake Forest School of Medicine, Winston-Salem, NC, USA.

^3^Bacterial Epidemiology and Antimicrobial Resistance Research Unit, U.S. Department of Agriculture Agricultural Research Service, Athens, Georgia, USA.

^4^Department of Biological Sciences, Florida State University, Tallahassee, Florida, USA.

**Running title:** Microbiome and antibiotic-resistant bacteria in retail shrimp.

***Corresponding author:**

Prashant Singh, PhD.

Assistance professor,

Department of Nutrition, Food and Exercise Sciences,

Florida State University, Tallahassee, FL 32306, USA.

Email: [psingh2@fsu.edu](mailto:psingh2@fsu.edu)

**Supplement Table 1**: Minimum inhibitory concentration of six gram-negative bacterial isolated from cooked (n=3) and raw (n=3) shrimp samples.

| *Morganella morganii* 31A-MNC | *Proteus mirabilis*  20B-MC1-1 | *Serratia marcescens* 10A-MNC | *Enterobacter hormaechei*  2B-MC1 | *Serratia marcescens* 28B-MC2 | *Vibrio paraheamolyticus*  24B-MC2 |
| --- | --- | --- | --- | --- | --- |
| Cooked | Cooked | Cooked | Raw | Raw | Raw |
|  | Farm raised | Farm raised | Farm raised | Wild caught |  |
| Vietnam | India | Indonesia | Ecuador | US | Panama |
| Resistant to | | | | | |
| Ampicillin Cefazolin | Ampicillin Cefazolin Cefuroxime Nitrofurantoin | Cefazolin Cefoxitin Cefuroxime Nitrofurantoin | Ampicillin Cefazolin Cefotetan Na Cefoxitin  Trimethoprim/ sulfamethoxazole | Ampicillin Cefazolin Cefoxitin Cefuroxime Ciprofloxacin Gatifloxacin  Nitrofurantoin | Cefazolin  Cefuroxime Nitrofurantoin |
| Intermediate Resistance | | | | | |
| Aztreonam  Cefuroxime  Imipenem  Nitrofurantoin | Aztreonam  Imipenem | Aztreonam  Imipenem | Ampicillin/ sulbactam  Aztreonam  Cefuroxime  Imipenem | Ampicillin/ sulbactam  Aztreonam  Imipenem | Ampicillin  Cefepime  Imipenem |
| MDR | MDR | MDR | MDR | MDR | MDR |

**Supplement FIG. 1.a.** The LEfSe analysis depicting relative abundance of unique taxonomic clades that were significantly different between the two groups of shrimps. The shrimps from US were uniquely distinguished by higher proportion of Proteobacteria*,* Alteromonadales, Rhizobiales, Synechococcaceae, Myxococcales and Planctomyces. **1.b.** PICRUSt analysis of the KEGG orthologs associated with bacterial clades. The shrimps from the US were characterized by higher (p < 0.01) abundance of bacterial taxa associated with the metabolism of amino acids, lipids, cofactors, vitamins and xenobiotic degradation.

Suppl. Fig. 1

(a)

(b)
